# Morphodynamics of submarine channel inception revealed by new experimental approach

**DOI:** 10.1038/ncomms10886

**Published:** 2016-03-21

**Authors:** Jan de Leeuw, Joris T. Eggenhuisen, Matthieu J. B. Cartigny

**Affiliations:** 1 Faculty of Geosciences, Utrecht University, PO Box 80021, 3508TA Utrecht, The Netherlands; 2 National Oceanography Centre, European Way, Southampton, Hampshire SO14 3ZH, UK

## Abstract

Submarine channels are ubiquitous on the seafloor and their inception and evolution is a result of dynamic interaction between turbidity currents and the evolving seafloor. However, the morphodynamic links between channel inception and flow dynamics have not yet been monitored in experiments and only in one instance on the modern seafloor. Previous experimental flows did not show channel inception, because flow conditions were not appropriately scaled to sustain suspended sediment transport. Here we introduce and apply new scaling constraints for similarity between natural and experimental turbidity currents. The scaled currents initiate a leveed channel from an initially featureless slope. Channelization commences with deposition of levees in some slope segments and erosion of a conduit in other segments. Channel relief and flow confinement increase progressively during subsequent flows. This morphodynamic evolution determines the architecture of submarine channel deposits in the stratigraphic record and efficiency of sediment bypass to the basin floor.

## Abstract (web summary)

Suspended sediment currents travel through channels on the ocean floor to deliver enormous volumes of sediment to the deep ocean. Here, using a new approach for scaled laboratory experiments, the authors show how feedback between these currents and their deposits drive the formation of these submarine channels.

## Graphical abstract

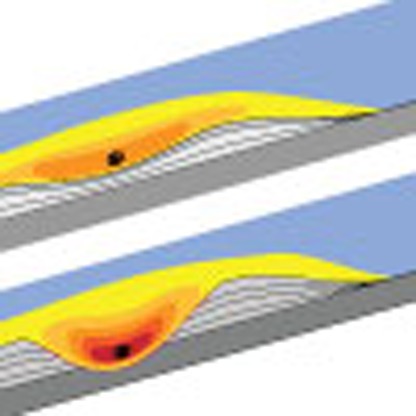

Extensive channelized seascapes have been revealed by seafloor surveys^1^,^2^,^3^,^4^. The channels are characterized by a continuous thalweg along which sediment-laden turbidity flows dominantly bypass sediment^5^. Submarine channels can be up to several kilometres wide and hundreds of kilometres long, and provide the transport pathways for large quantities of sediment, nutrients and carbon into the deeps of the world's ocean^1^,^6^, where the material is collected in basin-floor fans that form the largest sediment accumulations on the planet.

Seafloor and outcrop evidence demonstrates that channels are associated with erosion into underlying deposits^7^ and aggradation of deposits in levees, channel fills and splays^8^. Fundamentally, different causalities have been suggested in the spatial and temporal relations between erosive and depositional changes to the submarine landscape. Some studies^9^,^10^ envision an evolution where ‘only after an initial erosional phase and channel establishment are turbidity currents able to construct aggrading levees'^9^. This contrasts with suggestions that genetically linked precursor lobe morphologies may form an initial depositional template for subsequent channel incision^8^,^11^,^12^,^13^, and that channels may be entirely depositional both outside and inside the confining conduit^7^,^14^.

Subsurface and outcrop observations on channel morphology and channel deposits are static. Similarly, the presently available direct observations of active submarine channels^4^,^15^,^16^,^17^,^18^ do not span enough time to study morphodynamics of channel inception and evolution. In recent times, the extension of a submarine channel has been monitored in a quickly evolving system^19^. However, the data set did not provide direct information about the flow conditions during this morphological evolution. Therefore, modelling studies remain important in the investigation of the morphodynamic interplay between channel form and turbidity currents.

A limited number of experiments successfully produced subaqueous channels using a saline flow over a mobile substrate^20^,^21^,^22^. However, these flows could not produce depositional morphologies, as there was no suspended sediment load, which is vital for levee formation^22^. Therefore, these experiments provide limited insight into contributions of deposition and erosion during channel inception. Rowland *et al.*^23^ reviewed the full range of published numerical and physical experiments that have tried to achieve self-channelization^24^,^25^,^26^ by sediment-laden flows and concluded that channelization was not achieved in any of the cases.

We present experiments that for the first time capture self-channelization by turbidity currents. This was achieved by scaling sediment suspension in the experimental turbidity currents to the real world systems. This new scaling approach is called Shields scaling and focuses on two scaling parameters that regulate sediment suspension: (1) the Shields parameter and (2) a Reynolds scale of the sediment grains. The observed morphodynamic channel evolution establishes that channel inception can either commence with deposition of confining morphology by turbidity currents or erosion of a channel conduit. Thus, channel inception is not exclusively possible following erosion.

## Results

### Scaling approach

Classical turbidity current experiments^23^,^24^,^27^,^28^,^29^,^30^,^31^,^32^,^33^,^34^,^35^ have focused on two non-dimensional scaling characterizations of the fluid flow: the Froude number (*Fr*), which is the ratio between momentum and gravitational forces of the flow, and the Reynolds number (*Re*), characterizing the ratio between the momentum and the viscous forces that determine the turbulent state of the flow. As it is not possible to keep both *Fr* and *Re* equal to the natural analogues while scaling down flow size, it is common to keep the *Fr* similar to natural values and to only require a *Re* above the laminar-turbulent threshold^36^,^37^. This Froude scaling approach has proven to be valuable in understanding the flow dynamics of turbidity currents but it does not guarantee that flows are able to transport sediment in suspension.

Many Froude-scaled experiments displayed rapid sediment depletion and were therefore limited in clarifying patterns of deposition and erosion. Depletive flows rapidly lose their complete sediment load, because they do not have enough turbulent mixing to compensate for settling of sediment from suspension. To predict whether currents are able to entrain and transport sediment in suspension, it is important to consider the force ratios acting on the sediment grains. This leads to two additional constraints: the Shields parameter, being the ratio between the turbulent shear, as expressed by the shear velocity, and the gravity-induced settling^38^; and the particle *Re*, which is the ratio of grain size to the boundary layer thickness^39^. The former is more commonly quoted in turbidity current studies^29^ as the ratio between the shear velocity (u*) and the settling velocity (*u*_s_), but is here expressed as the Shields parameter. The latter is a Reynolds scale with significance for particle suspension near the bed. It describes the roughness of the sediment surface, which determines whether flow at the boundary is smooth and dominated by viscous forces, or rough and dominated by turbulent forces and shedding of turbulent eddies from particles at the bed surface^40^. If the boundary is smooth, a thin layer of laminar flow protects the bed and grains that settle into this near-bed boundary layer will no longer interact with suspending turbulent structures and are likely to remain deposited. In the transitionally rough regime, there is interaction of turbulent eddies with the bed but viscous forces also have a significant role. As experiments on channel inception are dependent on realistic turbulence–sediment interactions, both in the boundary layer and in suspension, it follows that such Shields scaling constrains must be satisfied.

**Figure 1:**
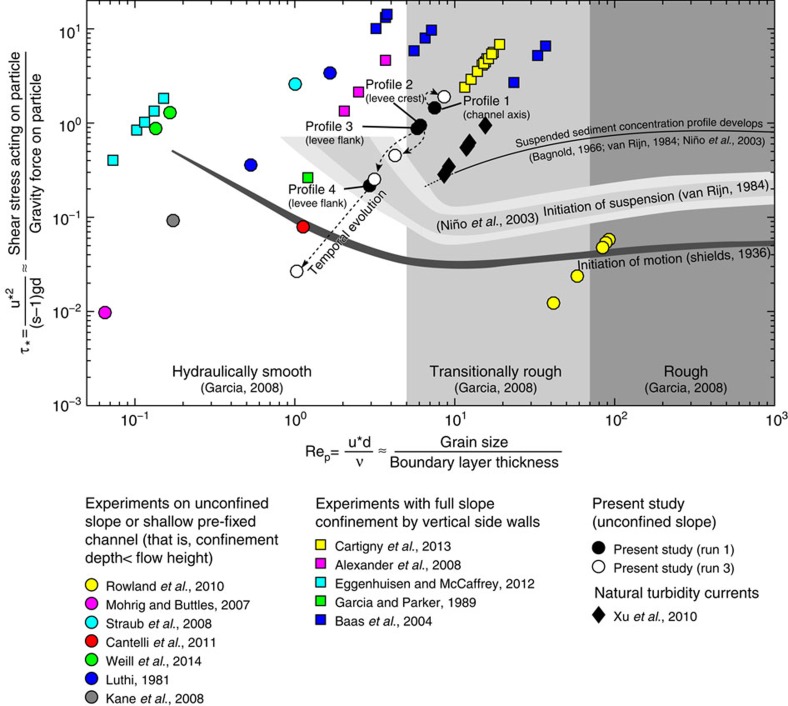
Regime diagram for sediment transport. Shields mobility diagram giving an overview of experimental conditions in previous studies^22^,^23^,^24^,^28^,^29^,^30^,^31^,^32^,^34^,^35^,^41^,^48^, natural flows^15^ and the experiments presented in this study. Regime boundaries based on refs 38, 40, 49, 50, 51.

The Shields scaling approach mirrors Froude scaling of the flow dynamics in the sense that one scale, namely the ratio of turbulent forces and gravity forces acting on the particle (the Shields parameter), is kept equal to real world values, whereas the other scale (the Reynolds particle scale) is relaxed, as long as rough to transitionally rough boundary layer conditions are maintained, to keep a realistic turbulent near-bed regime and aid sediment pick-up into suspension. These two scales form the axes of the classic Shields mobility diagram (Fig. 1), which enables a comparison between the present experiments, natural turbidity current conditions and previous experimental studies.

### Comparison with natural currents and previous experiments

*In situ* measurements of turbidity currents in the Monterey Canyon^15^,^16^ are used to estimate the position of a representative natural turbidity current on the Shields diagram (Supplementary Table 1 and Methods). The flows had a transitionally rough boundary and the Shields parameter plots above the suspension threshold (Fig. 1). Similarly, the boundary layer was transitionally rough in the present experiments and shear stresses were sufficiently high to support sustained suspension transport. As a result, these currents were sediment-bypass dominated along a significant part of the experimental domain. The experiments presented in this study were performed under Shields scaling conditions that are representative for the natural environment. Some previous confined slope experiments^24^,^41^ also plot in the natural turbidity current regime. The experiments presented here are, however, the first to satisfy both Froude and Shields scaling (Fig. 1) on an unconfined and erodible slope, making them suitable to study flow–substrate interactions during channel inception.

It emerges that many previous studies violated the proposed Shields scaling requirements, because the flows had smooth boundary layers and/or had shear stresses that were below the threshold for initiation of suspension. In the cases where flows had a smooth boundary layer and low Shields parameter^28^,^30^,^32^,^34^, flows were always depositional. Flows in other experiments^29^,^31^,^33^ had a higher Shields parameter but still a smooth boundary layer. Finally, the experiments of Rowland *et al.*^23^ fulfilled the roughness requirement; however, there the Shields parameter was only approximately equal to the critical value for initiation of bedload motion (Fig. 1). None of these experiments led to channelization morphodynamics.

### Morphological evolution

**Figure 2:**
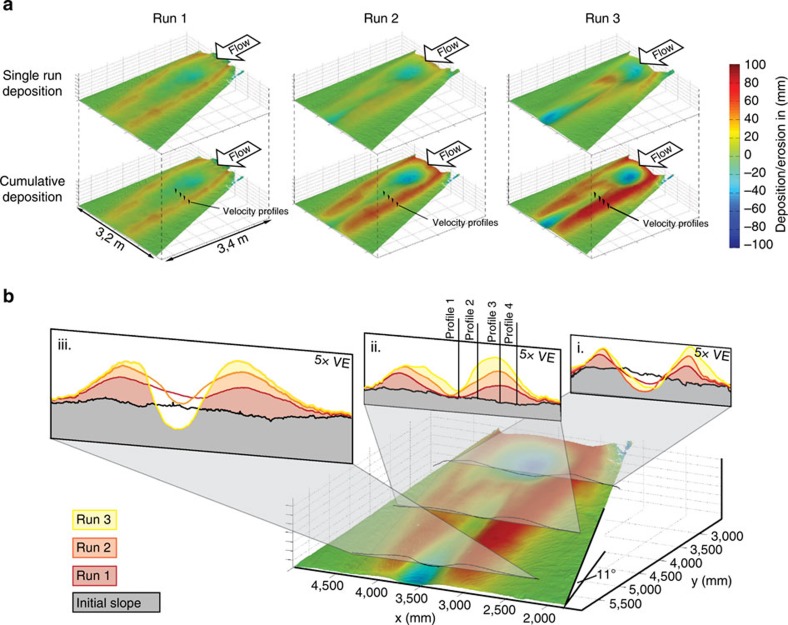
Maps of deposition and erosion. (**a**) Digital elevation models of the deposits formed by sandy turbidity currents. Colours indicate the thickness of the deposits/depth of erosion. Both the cumulative erosion/deposition and the erosion/deposition after each single run are shown. (**b**) DEM of the final deposit with cross-sections at the lower, middle and upper slope. Cross-sections have five times vertical exaggeration. Positions of the probes that measured downstream velocity profiles are indicated on cross-section ii.

Three turbidity currents with the same characteristics were released successively on a constant and initially featureless sand slope (boundary conditions in Table 1 and set-up shown in Supplementary Fig. 1). The first turbidity current deposited two sub-parallel ridges, while the flow largely bypassed in between the two ridges (Fig. 2). This pattern of deposition resulted in a morphology that confined the subsequent flows. The relief of this confinement was increased during the subsequent second and third run due to continued deposition on the ridges. A circular scour with a diameter of 70 cm and a final depth of 8 cm is created in between the levees on the upper slope domain throughout the three runs. This contributes to the channel relief in that reach. On the lower slope, erosion in between the ridges was only initiated during the second run. The ridges are built of layers deposited by successive turbidity currents that are thinning away from the channel axis and therefore the ridges can be qualified as levees^42^.

**Figure 3:**
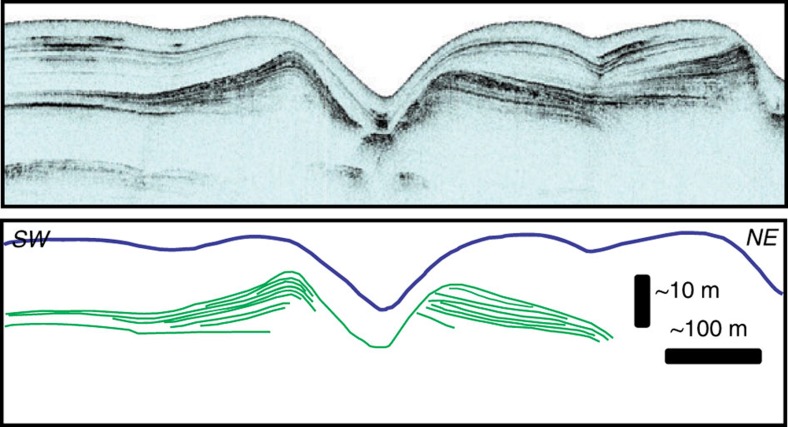
Example of a submarine channel on the modern seafloor. Chirp profile through a submarine channel that is part of the Lucia Chica channel system offshore central California^10^. The green lines indicate the turbidity current deposits. Compare this figure with the cross-sections of the experimental deposits in Fig. 2b. The blue line indicates the top of the hemipelagic drape (figure reprinted with permission from the publisher).

The cross-sectional geometry of the experimental channel compares well with submarine channels on the modern seafloor that have remained unfilled. The depth:width ratio of the Lucia Chica channel (Fig. 3) is 1:12, whereas the aspect ratio formed in the experiment varies between 1:9 and 1:23.

**Figure 4:**
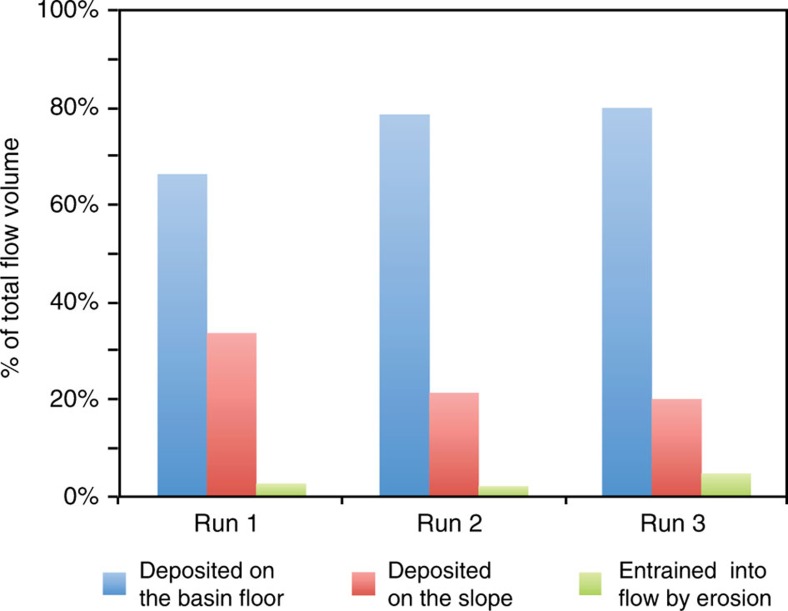
Volume of deposits on the slope. Fractions of the total sediment load of the flow deposited and eroded on the slope and on the basin floor. The volume of sediment supplied to the experiments is equal for each of the runs.

The amount of sediment bypass on the slope increases in each run as is indicated by an increasing fraction of the sediment reaching the base of slope. The fraction of sediment that reaches the base of slope increases from 66% in run 1 to 80% in run 3 (Fig. 4).

### Evolution of the flow field

**Figure 5:**
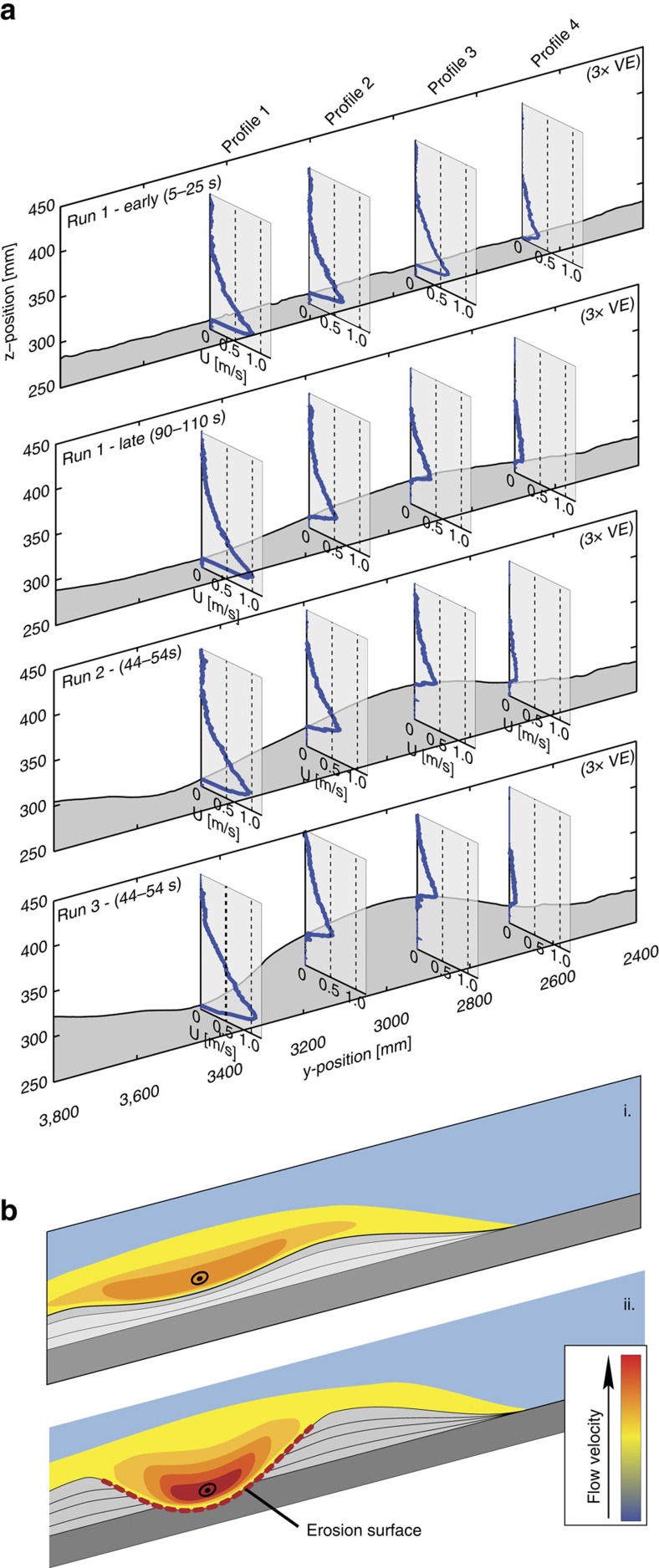
Changes in flow velocity as a result of increasing confinement. (**a**) Topographic profiles with time-averaged velocity profiles measured along the same transect at different time intervals (early run 1 (5–25 s), late run 1 (90–110 s), middle run 2 (44–54 s) and middle run 3 (44–54 s)). The full velocity time series are supplied in Supplementary Note 3. It is noteworthy that there is three times vertical exaggeration in the topographic profiles. (**b**) Model for the co-evolution of the flow field and the topography derived from the experiments: (i) broad and weakly confined flows build a subtle depositional confinement, because deposition rates are slightly lower in the axis. (ii) A threshold at which incision start is reached, causing a rapid increase in the confinement relief. It is noteworthy that the erosion of the channel floor was only observed downstream of the location of velocity profile 1.

The effect of the evolving topography on the flow field is shown by velocity profiles along a slope-perpendicular section (Fig. 5a and Supplementary Figs 2–4). At the beginning of run 1, when the slope was not yet modified, there was little across-flow variation in the downstream velocity profile. The confining morphology established by the end of run 1 resulted in an increase in flow velocity inside the confinement (*U*_max_ increases from 0.83 to 1.00 m s^−1^ at velocity profile 1; height of *U*_max_ was 1.2 cm) and a decrease in flow velocity outside of the confinement (*U*_max_ decreases from 0.64 to 0.38 m s^−1^ at velocity profile 3). It is noteworthy that the change in the flow field during run 1 was caused by a channel with a depth (*h*_*U*max_=2.6 cm) that was only a fraction of the flow height (*h*=7.3 cm). The increase in channel depth during runs 2 and 3 does not result in a systematic change in flow velocity at any of the profiling locations. These results confirm previous pre-fixed channel experiments^28^, which showed that a flow is already effectively confined within a conduit once the channel depth is greater than the height of the velocity maximum.

The spatial and temporal variations in flow velocity affected the ability of the flows to transport their sediment and these changes can be tracked within the Shields diagram (Fig. 1). The flow along the centreline of the slope (Fig. 5a, velocity profile 1) has a transitionally rough boundary layer and is able to bypass/erode sediment until the base of the slope. Flow at the off-axis locations of velocity profile 2 and 3 plot near the boundary between smooth and transitionally rough flow during run 1. The position of these points shifts towards the hydraulically smooth regime and below the suspension initiation threshold during run 3. Thus, the conditions at the locations of velocity profiles 2 and 3 are at or below the conditions for sediment bypass, and thus there is continuous deposition at these localities. The flow furthest away from the axis (Fig. 5a, velocity profile 4) plots within the field where flows have a smooth boundary layer and are below the boundary for suspended sediment transport during all runs. This indicates that flows that carry suspended load are highly depletive in these realms.

The temporal increase in axial flow velocity, which is caused by the progressively increasing confinement, causes an increase in the Shields parameter (Fig. 1, profile 1). The resulting small shift on the Shields diagram of the position of the flow at profile 1 appears to have little effect on the ability of the flow to transport sediment at this locality, because little deposition or erosion is observed here throughout the three runs. Although axial flow velocity was not monitored in the lower channel section, a larger shift on the Shields diagram can be inferred there because of the observed transition from deposition on the channel floor during run 1 to erosion on the channel floor during run 2 and 3 (Fig. 2b, cross-section iii.). The axial erosion is a further contributor to flow confinement in a dynamic feedback and consequently increases the rates of erosion.

In contrast, the flow conditions at the off-axis locations (velocity profiles 2, 3 and 4) are shifting in the Shields mobility diagram towards positions below the suspension initiation threshold and indeed there is continuous deposition at these localities.

In summary, spatial and temporal variation in the ability to transport sediment is predicted from the velocity measurements. The relative positions and temporal evolution on the Shields diagram predict the deposition of levees alongside a fairway dominated by sediment bypass and reflects progressive confinement increase during channel inception (Fig. 5b).

## Discussion

Significant debate has surrounded the nature of the relief that is created during the initial phase of channel formation. It has been argued that initial relief that turbidity currents create at a site of repeated activity is likely erosional^9^,^10^, which implies that levees commonly form from overspill after formation of an entrenched channel confinement. Trains of erosional scours are widely observed on the floors of channels on the modern ocean floor^3^,^4^,^9^,^42^,^43^,^44^ and are indeed a probable initial feature of channelization in many cases. Similarly, the scour in front of the outlet in the present experiments contributes to the initial confinement on the upper slope. However, the initial confinement along the middle and lower slope is created purely by depositional patterns arising from low deposition rates below the flow axis compared with the flow margins. Thus, the incipient levees formed by lateral variations in sediment transport processes and not by overspill from an already established channel. This morphodynamic development confirms the role that depositional templates may play in initial confinement^8^,^11^,^12^, and the experimental deposit cross-sections are strongly reminiscent of classic observational suggestions of depositional channel architecture^7^,^45^.

Channel axis erosion caused by initial depositional confinement represents transition from depositional channelization to erosional channelization (Fig. 5b). This is a confirmation of a ‘channelization threshold' at which a subtle confinement created by small depositional gradients causes incision, followed by a channelization feedback^11^. It emerges that channelization of turbidity currents can arise from both depositional and erosive sculpting of the seafloor, and may transition from depositional to erosive confinement. These flows thus have various intrinsic tendencies for channelization, which explains the ubiquitous presence of channels on submarine slopes.

The channel inception debate is just one of the many aspects of seafloor morphodynamics that can now be subjected to thorough testing in the laboratory environment. Previously, this was not possible, because experimental turbidity currents did not show realistic patterns of deposition and erosion as a result of inadequate scaling of the suspended sediment transport.

We conclude that confinement can progressively evolve from a depositional or erosional template, promoting gradual enhancement of sediment bypass on the slope. The increase in sediment bypass during the early phase of channel evolution will result in autogenic progradation of the system and deposits with according stacking patterns. Coarse-grained deposits can be expected at the base of finer-grained levees; such coupled stratigraphic bodies can be explained with a single genetic sequence of progressive channel inception, without the need to invoke changes in external mechanisms.

## Methods

### Description of the set-up

The experiments were conducted in the Eurotank Flume Laboratory at Utrecht University. The Eurotank measures 6 × 11 m in planform and was filled with water up to a level of 1.2 m above the horizontal floor (Supplementary Fig. 1). The bathymetry at the bottom of the tank consisted of a slope of 11° and a horizontal basin floor at the base of this slope. The slope was covered with a 10-cm-thick layer of loose sand that had the same grain-size distribution as the turbidity currents. A wooden duct was present at the top of the slope, to resemble a non-erodible canyon setting at the top of the slope. At the other end of the slope, a 10-cm-high ridge was placed, to provide down-dip accommodation for the sediment that reached the base of the slope.

### Sediment suspensions

Before each experiment, the sediment mixture was prepared outside the tank in a 1.1-m^3^ mixing tank, with two propellers designed to homogenize sediment–water mixtures up to 30% volumetric sediment concentrations. The quartz sand used to make the suspensions had a median grain size (*D*_50_) of 141 μm, a *D*_10_ of 44 μm and a *D*_90_ of 199 μm (Supplementary Fig. 5), and had a specific density of 2,650 kg m^−3^. The grain size was analysed using a Malvern Mastersizer particle sizer.

### Data collection

During the experiments, a slurry pump was used to supply the suspension to the set-up. A discharge meter (Krohne Optiflux 2300) was mounted in the supply pipe. The discharge was regulated by a Labview control system that adjusted the pump speed whenever the measured discharge deviated from a set reference value. The discharge during each of the experiments presented here was 30 m^3^ h^−1^. The experiments lasted ∼100 s before the mixing tank was drained. Four Ultrasonic Doppler Velocity Profiler probes (UVP Duo MX, 1 MHz) were installed on an aluminium frame, to monitor the flow field during the experiments. These probes were set up at around 0.15 m above the erodible basin floor, with their beam pointing diagonally down into the flow at an angle of 60° relative to the initial local slope of the flume floor. The planview location of the probes is indicated in Fig. 2. Each of these probes measured a full profile of bed-parallel flow in the direction of the probe orientation. The profiles had a spatial resolution of 0.64 mm and the measurement frequency was 1.81 Hz. Individual velocity profiles have a spiky appearance due to the turbulent nature of the flows. Therefore, time-averaging was applied to create smoother profiles as presented in Fig. 5a. After each experimental run, the basin was drained to expose the deposit. Next, a digital elevation model (DEM) with a horizontal resolution of 2 × 2 mm was created using a laser scanner. By subtracting the DEM of the experimental deposits and a DEM of the sediment bed before the experiments, a map of deposition and erosion was created for each experiment.

### Determination of flow conditions

The following flow parameters were required to determine the position of each reviewed experiment on the Shields diagram (Fig. 1):

Grain size (*d*): Here, the median grain size of the initial sediment mixture was used.

Kinematic viscosity (*v*): Here, the viscosity of clear water at 20° was used (1 × 10^−6^).

Shear velocity (U*): When estimates are supplied in experimental studies, then they are followed. Elsewise, the shear velocities were determined as^46^ 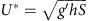.

For the present experiments, the shear velocity was determined using^39^:


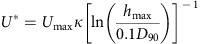


where *h*_max_ is the height of the velocity maximum and *U*_max_ is the maximum velocity.

Reduced gravity (*g*′): *g*(Δ*ρ*/*ρ*), where *ρ* is the density of the suspension and Δ*ρ* is the excess density of the sediment submerged in the ambient fluid. To calculate the density of the suspension, it is assumed that the density of the sediment concentration is equal to the concentration of the initial mix.

Flow height up to the velocity maximum (*h*): If only the total flow thickness was given, it was assumed that the height of the velocity maximum is at one-fourth of the total flow thickness.

Bed slope (*S*): sin(bed slope in degrees).

Flow conditions for turbidity currents in the Monterey canyon were determined using information reported in ref. 15. A representative median grain size for the turbidity currents was estimated from sediment cores of the Monterey canyon floor. Core data in ref. 47 shows that a broad range of grain sizes (ranging from silt to boulders) were deposited on the canyon floor. Middle sand (diameter of 350 μm) was chosen as a representative grain size, because it was the most common grain size in the cores. The *D*_90_ was estimated at 500 μm. The shear velocity was determined using the formula that is also used for this purpose for the present experiments.

## Additional information

**How to cite this article:** de Leeuw, J. *et al.* Morphodynamics of submarine channel inception revealed by new experimental approach. *Nat. Commun.* 7:10886 doi: 10.1038/ncomms10886 (2016).

## Supplementary Material

Supplementary Information Supplementary Figures 1-5 and Supplementary Table 1

## Figures and Tables

**Table 1:** Boundary conditions of the experiments.

Input sediment concentration	17% vol
Suspension discharge	30 m^3^ h^−1^
Median grain size	141 μm
Bed slope	11°
