# Supplementary material for: Morphodynamics of submarine channel inception revealed by new experimental approach
Source: Nat Commun. 2016 Mar 21;7:10886. doi: 10.1038/ncomms10886 (PMC4802116; doi:10.1038/ncomms10886)
Supplement: Supplementary Information — Supplementary Figures 1-5 and Supplementary Table 1 [file ncomms10886-s1.pdf]

Supplementary figure 1

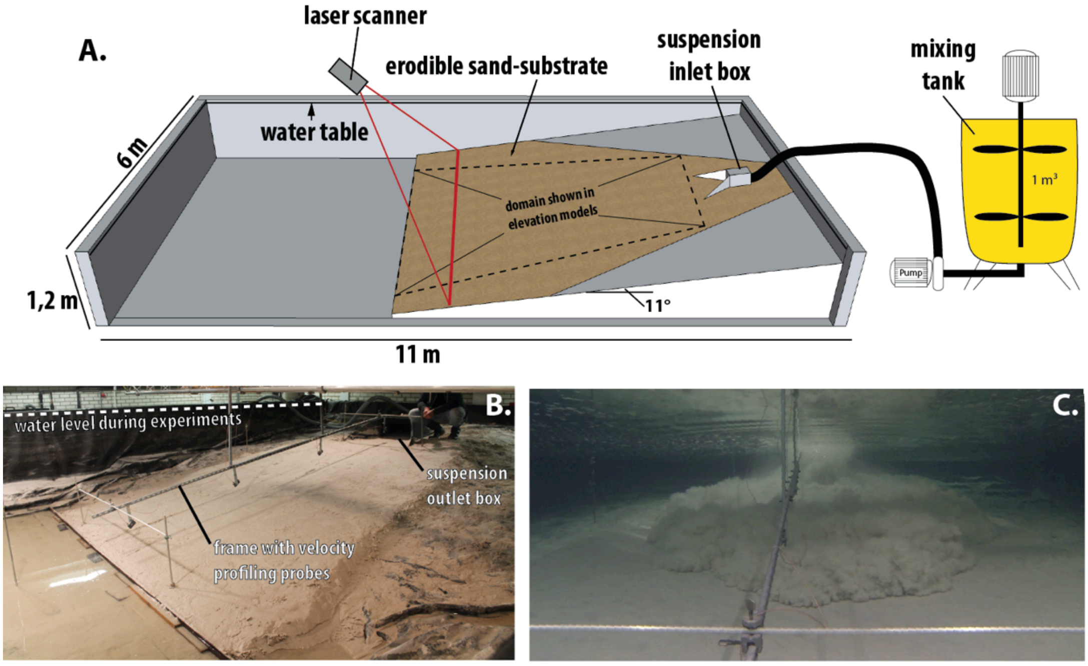

Experimental setup details. *A. Schematic overview of the experimental set-up. B. Drained set-up prior to an experiment. C. Underwater view showing a turbidity current flowing downslope during an experiment.*

Supplementary figure 2

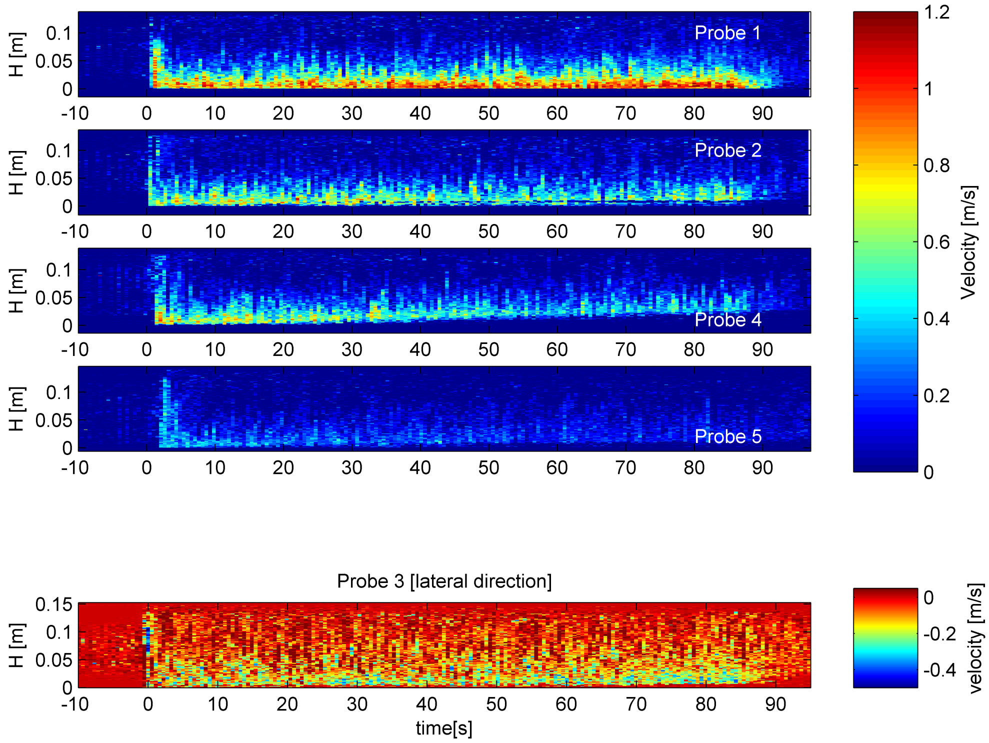

UVP velocity time-series of run 1

Supplementary figure 3

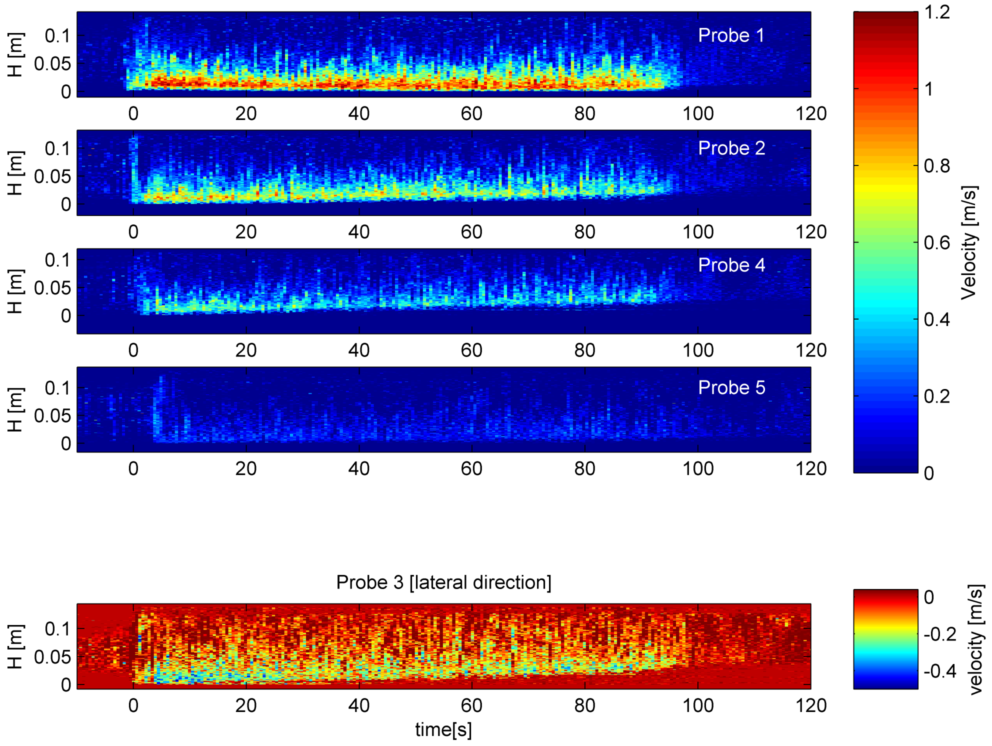

UVP velocity time-series of run 2

Supplementary figure 4

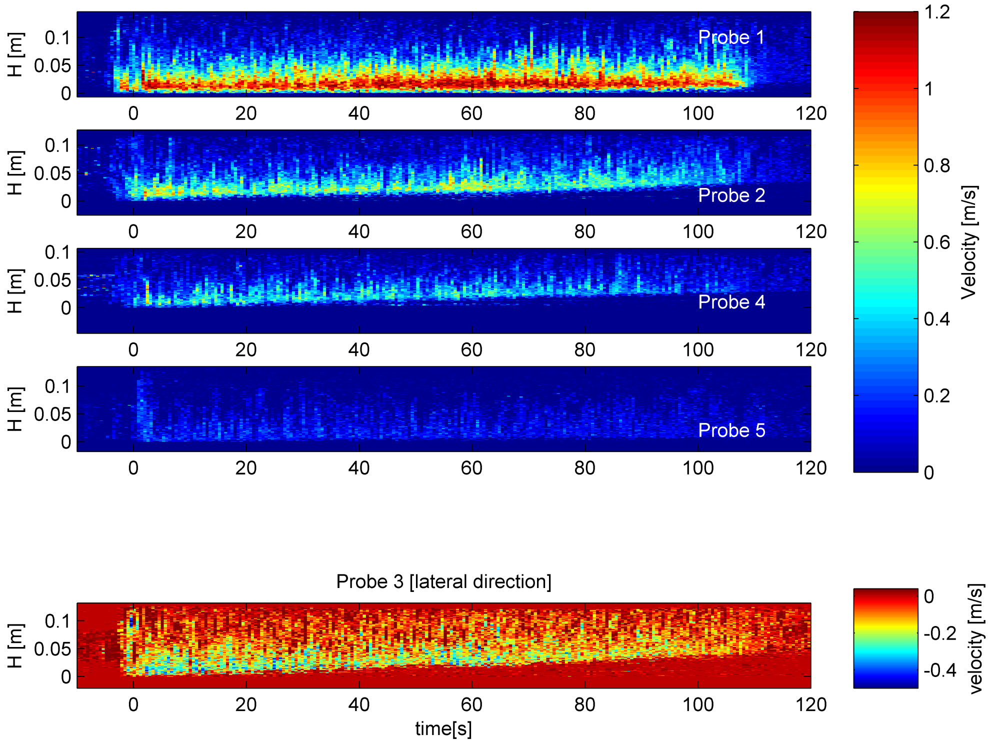

UVP velocity time-series for run 3.

Supplementary figure 5

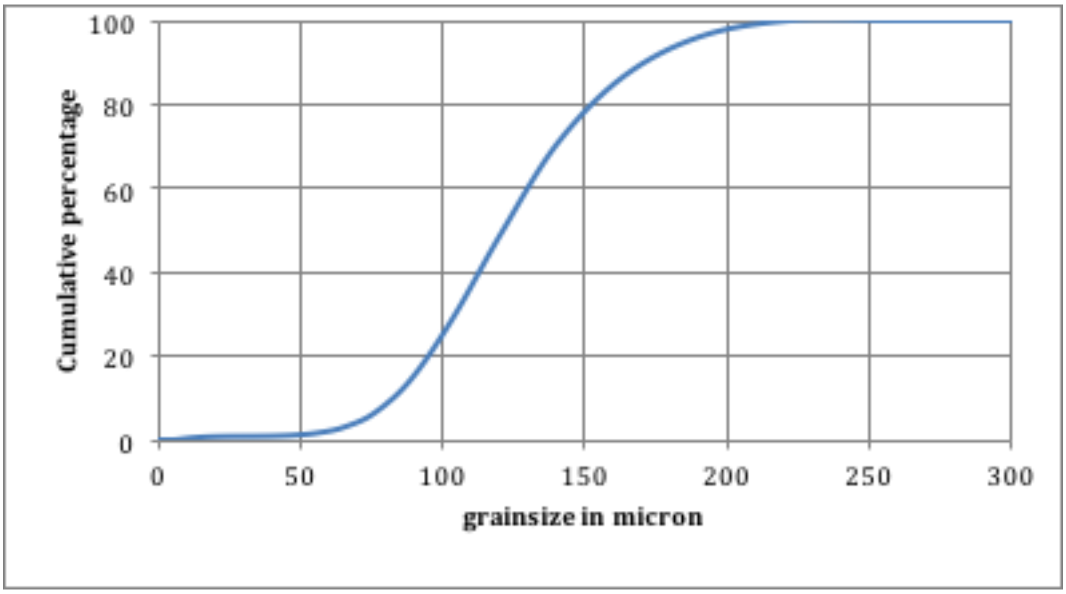

Grain size distribution of the sediment used. *The sediment is used both for the suspensions and for the sediment bed in the set-up.*

Supplementary table 1

Calculate T* and Re p* values

author + year	run #	Cvol	RHO sed	g' (submerged gravity)	R (hydraulic radius)	S (slope)	S (density in kg/dm3)	d (median grain size)	u*	T*	Re p*
Rowland 2010	1 (baseline)	(saline currents)	1200	0.00E+00	1.67E-02	0.006	1.2	0.0042	0.00E+00	0.00E+00	0.00E+00
Rowland 2010	2 (baseline)	(saline currents)	1200	0.00E+00	1.67E-02	0.006	1.2	0.0042	0.00E+00	0.00E+00	0.00E+00
Rowland 2010	3	(saline currents)	1200	7.00E-02	1.67E-02	0.006	1.2	0.0042	2.20E-02	5.88E-02	9.20E+01
Rowland 2010	4	(saline currents)	1200	1.80E-01	1.67E-02	0.006	1.2	0.0042	2.10E-02	5.36E-02	8.78E+01
Rowland 2010	5	(saline currents)	1200	3.40E-01	1.67E-02	0.006	1.2	0.0042	2.00E-02	4.86E-02	8.37E+01
Rowland 2010	6	(saline currents)	1200	5.50E-01	1.67E-02	0.006	1.2	0.0042	1.40E-02	2.38E-02	5.86E+01
Rowland 2010	7	(saline currents)	1200	1.20E+00	1.67E-02	0.006	1.2	0.0042	1.00E-02	1.21E-02	4.18E+01
Cartigny 2014	C09-S09-A	0.09	2650	1.46E+00	2.57E-02	0.156	2.65	0.00015	7.66E-02	2.42E+00	1.14E+01
Cartigny 2014	C09-S11-A	0.09	2650	1.46E+00	2.59E-02	0.191	2.65	0.00015	8.49E-02	2.97E+00	1.27E+01
Cartigny 2014	C13-S11-A	0.13	2650	2.10E+00	2.47E-02	0.191	2.65	0.00015	9.96E-02	4.09E+00	1.49E+01
Cartigny 2014	C13-S12-N	0.13	2650	2.10E+00	2.43E-02	0.208	2.65	0.00015	1.03E-01	4.39E+00	1.54E+01
Cartigny 2014	C15-S10-A	0.15	2650	2.43E+00	2.46E-02	0.174	2.65	0.00015	1.02E-01	4.28E+00	1.52E+01
Cartigny 2014	C15-S11-N	0.15	2650	2.43E+00	2.50E-02	0.191	2.65	0.00015	1.08E-01	4.77E+00	1.61E+01
Cartigny 2014	C17-S07-A	0.17	2650	2.75E+00	2.56E-02	0.122	2.65	0.00015	9.27E-02	3.55E+00	1.39E+01
Cartigny 2014	C17-S09-N	0.17	2650	2.75E+00	2.43E-02	0.156	2.65	0.00015	1.02E-01	4.30E+00	1.53E+01
Cartigny 2014	C21-S08-A	0.21	2650	3.40E+00	2.52E-02	0.139	2.65	0.00015	1.09E-01	4.92E+00	1.63E+01
Cartigny 2014	C21-S09-N	0.21	2650	3.40E+00	2.53E-02	0.156	2.65	0.00015	1.16E-01	5.54E+00	1.73E+01
Cartigny 2014	C26-S07-A	0.26	2650	4.21E+00	2.58E-02	0.122	2.65	0.00015	1.15E-01	5.45E+00	1.72E+01
Cartigny 2014	C26-S09-N	0.26	2650	4.21E+00	2.51E-02	0.156	2.65	0.00015	1.29E-01	6.82E+00	1.92E+01
Alexander 2008	H0500	0.012	2500	1.77E-01	5.38E-02	0.000	2.5	0.000059	0.00E+00	0.00E+00	0.00E+00

Alexander 2008	H0306	0.012	2500	1.77E-01	6.38E-02	0.105	2.5	0.000059	3.44E-02	1.37E+00	2.02E+00
Alexander 2008	H0909	0.012	2500	1.77E-01	6.70E-02	0.156	2.5	0.000059	4.30E-02	2.13E+00	2.53E+00
Alexander 2008	H0620	0.012	2500	1.77E-01	6.54E-02	0.364	2.5	0.000059	6.48E-02	4.85E+00	3.81E+00
Eggenhuisen 2012	1	0.012121212	2650	8.93E-02	1.40E-01	0.017	2.65	0.0000093	7.80E-03	4.05E-01	7.23E-02
Eggenhuisen 2012	2	0.024242424	2650	1.28E-01	1.10E-01	0.017	2.65	0.0000093	1.12E-02	8.34E-01	1.04E-01
Eggenhuisen 2012	3	0.048484848	2650	1.88E-01	8.00E-02	0.017	2.65	0.0000093	1.24E-02	1.02E+00	1.04E-01
Eggenhuisen 2012	4	0.036363636	2650	2.71E-01	7.00E-02	0.017	2.65	0.0000093	1.42E-02	1.34E+00	1.32E-01
Eggenhuisen 2012	5	0.060606061	2650	3.01E-01	1.00E-01	0.017	2.65	0.0000093	1.65E-02	1.81E+00	1.53E-01
Mohrig 2007	1	0.008	2650	1.29E-01	1.50E-02	0.002	2.65	0.00003	2.16E-03	9.61E-03	6.45E-02
Straub et al. 2008	1	0.004242424	2650	1.96E-01	1.00E-01	0.000	2.65	0.000029	1.40E-02	4.18E-01	4.04E-01
Cantelli 2011		0.02	2650	3.24E-01	5.00E-03	0.080	2.65	0.0001	1.14E-02	8.01E-02	1.13E+00
Weill, 2014	5	(saline currents)	1040	7.41E-01	2.00E-04	0.139	1.08	0.00003	4.54E-03	8.77E-01	1.36E-01
Weill, 2014	6	(saline currents)	1040	7.41E-01	2.00E-04	0.139	1.08	0.00003	4.54E-03	8.77E-01	1.36E-01
Weill, 2014	7	(saline currents)	1040	7.41E-01	2.00E-04	0.139	1.08	0.00003	4.54E-03	8.77E-01	1.36E-01
Weill, 2014	10	(saline currents)	1040	7.41E-01	2.00E-04	0.208	1.08	0.00003	5.55E-03	1.31E+00	1.66E-01
Weill, 2014	11	(saline currents)	1040	7.41E-01	2.00E-04	0.208	1.08	0.00003	5.55E-03	1.31E+00	1.66E-01
Luthi, 1981	1	0.004242424	2650	6.48E-02	3.75E-02	0.087	2.65	0.000037	1.46E-02	3.54E-01	5.36E-01
Luthi, 1981	3	0.040606061	2650	6.20E-01	3.75E-02	0.087	2.65	0.000037	4.50E-02	3.39E+00	1.66E+00
kane, 2008		0.05	2650	8.09E-01	7.49E-02	0.000	2.65	0.0000273	6.38E-03	9.21E-02	1.73E-01
Garcia, 1993	T3	(saline currents)	1350	1.50E-01	2.50E-02	0.080	1.35	0.0001	1.55E-03	7.00E-03	1.54E-01
Garcia, 1993	A1	(saline currents)	1350	1.50E-01	2.50E-02	0.080	1.35	0.0001	1.81E-03	9.55E-03	1.80E-01
Garcia, 1993	A2	(saline currents)	1350	1.50E-01	2.50E-02	0.080	1.35	0.0001	1.61E-03	7.56E-03	1.60E-01
Garcia, 1993	A3	(saline currents)	1350	1.70E-01	2.50E-02	0.080	1.35	0.0001	1.61E-03	7.56E-03	1.60E-01
Garcia, 1993	A4	(saline currents)	1350	2.50E-01	2.50E-02	0.080	1.35	0.0001	2.08E-03	1.26E-02	2.07E-01
Garcia, 1993	B2	(saline currents)	1350	2.50E-01	2.50E-02	0.080	1.35	0.00018	2.27E-03	8.35E-03	4.07E-01
Garcia, 1993	B3	(saline currents)	1350	2.10E-01	2.50E-02	0.080	1.35	0.00018	1.84E-03	5.48E-03	3.30E-01
Garcia, 1993	B5	(saline currents)	1350	2.50E-01	2.50E-02	0.080	1.35	0.00018	2.26E-03	8.27E-03	4.05E-01

Garcia, 1993	B6	(saline currents)	1350	2.10E-01	2.50E-02	0.080	1.35	0.00018	2.04E-03	6.74E-03	3.66E-01
Garcia, 1993	C1	(saline currents)	1350	2.20E-01	2.50E-02	0.080	1.35	0.00018	2.07E-03	6.94E-03	3.71E-01
Garcia, 1993	C3	(saline currents)	1350	2.00E-01	2.50E-02	0.080	1.35	0.00018	1.94E-03	6.10E-03	3.48E-01
Garcia, 1993	D1	(saline currents)	1350	1.70E-01	2.50E-02	0.080	1.35	0.00018	1.83E-03	5.42E-03	3.28E-01
present experiments	R49 probe 1	0.11	2650	1.78E+00	7.00E-02	0.191	2.65	0.000135	4.52E-02	9.34E-01	6.07E+00
present experiments	R49 probe 2	0.11	2650	1.78E+00	7.00E-02	0.191	2.65	0.000135	5.59E-02	1.43E+00	7.51E+00
present experiments	R49 probe 3	0.11	2650	1.78E+00	7.00E-02	0.191	2.65	0.000135	4.34E-02	8.63E-01	5.84E+00
present experiments	R49 probe 4	0.11	2650	1.78E+00	7.00E-02	0.191	2.65	0.000135	2.17E-02	2.15E-01	2.91E+00
present experiments	R51 probe 1	0.11	2650	1.78E+00	7.00E-02	0.191	2.65	0.000135	3.14E-02	4.51E-01	4.22E+00
present experiments	R51 probe 2	0.11	2650	1.78E+00	7.00E-02	0.191	2.65	0.000135	6.39E-02	1.87E+00	8.59E+00
present experiments	R51 probe 3	0.11	2650	1.78E+00	7.00E-02	0.191	2.65	0.000135	2.35E-02	2.52E-01	3.16E+00
present experiments	R51 probe 4	0.11	2650	1.78E+00	7.00E-02	0.191	2.65	0.000135	7.62E-03	2.66E-02	1.02E+00
Baas, 2004	1	0.27	2650	4.37E+00	5.50E-02	0.150	2.65	0.000235	1.90E-01	9.46E+00	4.44E+01
Baas, 2004	2	0.27	2650	4.37E+00	5.50E-02	0.150	2.65	0.000235	1.90E-01	9.46E+00	4.44E+01
Baas, 2004	3	0.35	2650	5.67E+00	5.50E-02	0.150	2.65	0.000235	2.16E-01	1.23E+01	5.05E+01
Baas, 2004	4	0.14	2650	2.27E+00	5.50E-02	0.150	2.65	0.000235	1.37E-01	4.90E+00	3.20E+01
Baas, 2004	7	0.27	2650	4.37E+00	5.50E-02	0.065	2.65	0.00004	1.25E-01	2.40E+01	4.96E+00
Baas, 2004	8	0.21	2650	3.40E+00	5.50E-02	0.065	2.65	0.00004	1.10E-01	1.87E+01	4.38E+00
Baas, 2004	9	0.29	2650	4.69E+00	5.50E-02	0.065	2.65	0.00004	1.29E-01	2.58E+01	5.14E+00
Baas, 2004	10	0.29	2650	4.69E+00	5.50E-02	0.065	2.65	0.00004	1.29E-01	2.58E+01	5.14E+00
Baas, 2004	11	0.29	2650	4.69E+00	5.50E-02	0.065	2.65	0.00004	1.29E-01	2.58E+01	5.14E+00
Baas, 2004	12	0.29	2650	4.69E+00	5.50E-02	0.065	2.65	0.000069	1.29E-01	1.49E+01	8.87E+00
Baas, 2004	13	0.35	2650	5.67E+00	5.50E-02	0.065	2.65	0.000069	1.42E-01	1.80E+01	9.75E+00
Baas, 2004	14	0.21	2650	3.40E+00	5.50E-02	0.065	2.65	0.000069	1.10E-01	1.08E+01	7.55E+00
Garcia, 1989	min			2.00E-02	2.50E-02	0.080	2.5	0.000005	6.33E-03	5.46E-01	3.15E-02
Garcia, 1989	max	0.01	2650	0.15	2.50E-02	0.080	2.65	0.00007	1.73E-02	2.66E-01	1.21E+00
Gray, 2005	3 deg slope	0.01	2500	1.47E-01	2.00E-02	0.052	2.5	0.000071	1.24E-02	1.48E-01	8.78E-01
Gray, 2005	6 deg slope	0.01	2500	1.47E-01	2.00E-02	0.105	2.5	0.000071	1.75E-02	2.95E-01	1.24E+00
Gray, 2005	9 deg slope	0.01	2500	1.47E-01	2.00E-02	0.156	2.5	0.000071	2.15E-02	4.41E-01	1.52E+00

yu, 2011	silica 1	0.02	2650	3.24E-01	1.00E-02	0.191	2.65	0.00005	2.49E-02	7.64E-01	1.24E+00
yu, 2011	silica 2	0.02	2650	3.24E-01	1.00E-02	0.122	2.65	0.00002	1.99E-02	1.22E+00	3.96E-01
yu, 2011	silica 3	0.02	2650	3.24E-01	1.00E-02	0.061	2.65	0.0000083	1.41E-02	1.47E+00	1.16E-01
Felix, 2005	kaolin	0.14	2650	2.27E+00	3.75E-02	0.087	2.65	0.000009	8.61E-02	5.09E+01	7.71E-01
Felix, 2005	silica flour	0.28	2650	4.53E+00	3.75E-02	0.087	2.65	0.000006	1.22E-01	1.53E+02	7.27E-01
Straub & Mohrig 2008							2.65	0.000029	3.50E-02	2.61E+00	1.01E+00
Xu et al., 2010	2 @ 820 m						2.65	0.00035	4.83E-02	4.12E-01	1.68E+01
Xu et al., 2010	2 @ 1020 m						2.65	0.00025	4.71E-02	5.49E-01	1.17E+01
Xu et al., 2010	2 @ 1445 m						2.65	0.00025	6.17E-02	9.43E-01	1.54E+01
Xu et al., 2010	3 @ 820 m						2.65	0.00025	4.97E-02	6.11E-01	1.24E+01
Xu et al., 2010	3 @ 1020 m						2.65	0.00025	0.034113695	2.88E-01	8.49E+00
Xu et al., 2010	4 @ 1020 m						2.65	0.00025	0.048254738	5.76E-01	1.20E+01
Xu et al., 2010	4 @ 1445 m						2.65	0.00025	0.037143294	3.41E-01	9.25E+00
